# The Effect of Hybrid Treatment Combining Boriding and Nanobainitising on the Tribological and Mechanical Properties of 66SiMnCrMo6-6-4 Bearing Steel

**DOI:** 10.3390/ma16093436

**Published:** 2023-04-28

**Authors:** Grzegorz Łukaszewicz, Michał Tacikowski, Michał Kulka, Krzysztof Chmielarz, Wiesław A. Świątnicki

**Affiliations:** 1 Faculty of Materials Science and Engineering, Warsaw University of Technology, ul. Wołoska 141, 02-507 Warsaw, Poland; michal.tacikowski@pw.edu.pl (M.T.); krzysztof.chmielarz.dokt@pw.edu.pl (K.C.); wieslaw.swiatnicki@pw.edu.pl (W.A.Ś.); 2 Faculty of Materials Engineering and Technical Physics, Poznan University of Technology, Pl. M. Skłodowskiej-Curie 5, 60-965 Poznan, Poland; michal.kulka@put.poznan.pl

**Keywords:** hybrid treatment, pack boriding, nanobainitic steel, wear

## Abstract

The effect of a new hybrid heat treatment consisting of pack-boriding and nanobainitising on the microstructure and properties of EN 66SiMnCrMo6-6-4 bearing steel was investigated. The hybrid treatment produces a new high-strength (ca. 1480 MPa) material with a hard boride (ca. 2000 HV0.05) surface layer and a relatively ductile nanobainitic core. The formation of the boride layer significantly improves wear resistance. The boride layer, which is hard but susceptible to cracking, reduces the mechanical properties under tensile and impact loads. However, the borided and nanobainitised steel exhibits much higher tensile strength and ductility and slightly better impact toughness than steel after post-boriding quenching and tempering.

## 1. Introduction

Boriding is a thermo-chemical treatment that leads to the formation of an iron boride layer on the steel surface. As a result, the steel surface acquires high wear and corrosion resistance [1,2]. In particular, boriding ensures high wear resistance in dry friction conditions, which makes it suitable for heavy-duty applications. For this reason, borided layers are used as a cover for various tools and machine parts, especially in the mining industry. The measurable effect of their application is the extended life of working tools, resulting in economic and environmental benefits [1,3].

During boriding, the steel surface undergoes saturation with boron atoms. This results in the development of a boride layer. Its properties depend on the boriding technology, process conditions and the steel’s chemical composition [4]. For this reason, a lot of research has focused on the recognition and optimisation of boriding processes [5]. A large number of studies are devoted to creating models of the kinetics of boriding. They aim to predict the growth of the boride layer depending on the method, temperature and time of the process [6,7,8]. Other studies considered optimising the boriding environment (container design, boriding medium) [9,10,11]. Furthermore, methods in which auxiliary processes precede [12,13,14] or follow boriding [15,16,17] were studied. These studies are essential because they help increase the treatment’s benefits.

However, previous research focused on the structure and properties of the produced layers without paying much attention to the steel core issue. For many applications, only the surface performance is critical; the core’s durability is equally important for many others. Even if the bulk material properties are not crucial in a given application, it is still important to understand the interactions between the steel core and surface layer.

Conventional heat treatment of borided steel includes quenching and tempering or isothermal quenching [1]. Boriding and austenitising can be carried out in one segment or separately. It should be noted that over the past four decades, new types of heat treatments have been developed. Of particular interest is the process of isothermal hardening in the lower temperature range of the bainitic transformation, leading to the formation of nanocrystalline bainite (called nanobainite) [18]. It should be emphasised that nanobainite differs from conventional bainitic structures. The first distinguishing feature concerns phase composition. Nanobainite contains, apart from ferrite, only residual austenite. No precipitations of cementite are present in the microstructure, as in the case of conventional bainite. The cementite precipitation is blocked through the appropriate additions of silicon and aluminium [19,20]. Moreover, the structural constituents—the plates of bainitic ferrite and the films of residual austenite—have a thickness smaller than 100 nm. The requirement to obtain a nanostructure is both the suitable chemical composition of the steel and the proper heat treatment conditions. Nanobainitic steels exhibit excellent mechanical properties. They can exhibit tensile strength higher than 2000 MPa while maintaining a high level of plasticity and fracture toughness [21,22,23,24].

Combining the advantages of boriding and nanobainitising of steel appears worth investigating. In recent years, several research studies dealing with boriding and bainitising have been carried out [25,26,27]; however, they did not concern a carbide-free nanobainite. In addition, these studies focused mainly on the properties of the boride layer. They did not investigate the effect of boriding on the mechanical properties of the layer-core system. The present work aims to provide knowledge in this area and exhibit the results of hitherto unknown hybrid treatments. This work also aims to determine the effect of nanobainitising on the microstructure and properties of the pack-borided bearing steel, which, to the authors’ knowledge, has not been carried out so far.

## 2. Materials and Methods

### 2.1. Substrate Material

In this research, EN 66SiMnCrMo6-6-4 bearing steel was used. Its chemical composition is given in Table 1. The amounts of carbon and other alloying elements were advantageous from the point of view of low-temperature bainitising. In particular, the contribution of silicon was beneficial for impeding carbides precipitation and, thus, favourable for carbide-free nanobainite formation.

### 2.2. Pack Boriding Process

**Figure 1:**
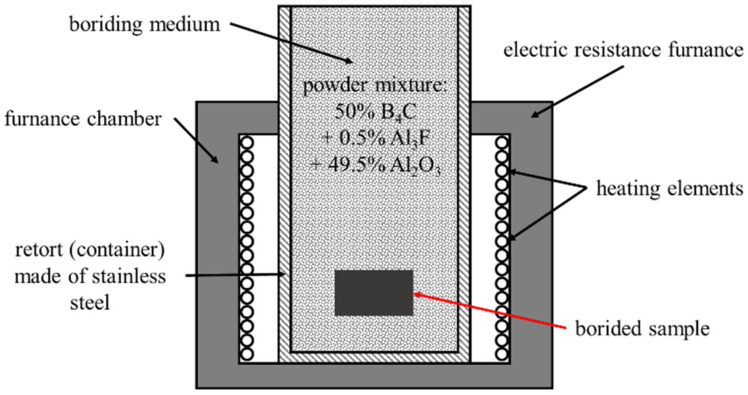
Scheme of pack boriding using open retort placed in a typical electric furnace.

For pack boriding, various powder mixtures were used. Recently, powder mixtures with boron carbide (B_4_C) as a boron source have been intensively developed. The effectiveness of boriding with only B_4_C was too low. Commercial powder mixtures, such as EKABOR^®^ and DURBORID^®^, were specially prepared based on boron carbide. EKABOR^®^ I, II and III were characterised by approximately the same chemical composition, consisting of B_4_C as a boron source, KBF_4_ as an activator and SiC as a diluent. In the present study, due to the significant concentration of silicon (1.625 wt%) in the steel used, another powder mixture (without SiC) was used to diminish the effect of silicon concentration on the microstructure of the produced boride layers. A powder mixture of 50% B_4_C (a boron source), 0.5% AlF_3_ (an activator) and 49.5% Al_2_O_3_ (a diluent) was used as a boriding medium. The pack boriding of the samples was carried out in an open retort filled with boriding powder and placed in a typical electric furnace without an additional seal according to the scheme shown in Figure 1. Such a boriding technique was described in the monograph [5] in detail. The retort was put into the furnace chamber in such a way that the upper part of the retort extended outside the furnace. The boriding powder in the upper part of the retort (outside the furnace) became its natural seal. The temperature of the surface of the boriding mixture was so low that this powder mixture did not oxidise. The gases created in the bottom part of the retort re-sublimated in its cold upper part and, therefore, did not move outside the retort. After the process, the retort with the samples within was removed from the furnace chamber and cooled in the air. The samples were removed from the retort after the retort had cooled to ambient temperature in order to avoid oxidation.

### 2.3. Post-Boriding Heat Treatments

**Figure 2:**
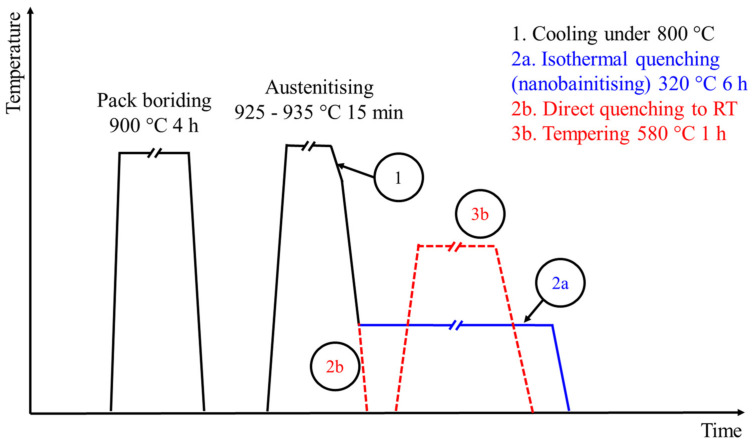
Scheme of hybrid heat treatments: boriding with nanobainitising and boriding with quenching and tempering.

After boriding, the samples were subjected to two different heat treatments. The first set of samples was subjected to a nanobainitising process, while the second set of samples was subjected to quenching and tempering. In both cases, the austenitising segment proceeded in the same way. The samples were treated in the vacuum tube furnace. Firstly, samples were heated in a furnace to austenitising temperature. After the batch reached a temperature of 925 °C, the samples were heated for 15 min to about 935 °C. The samples were then moved to the cold zone of the furnace, where they were cooled to a temperature of 800 °C. Afterwards, they were transferred from the furnace and subjected to subsequent heat treatment segments. This course of austenitising in a vacuum was necessary to prevent the oxidation of the borides layer at temperatures above 800 °C. In the case of the first variant (hereinafter called Br-NB), the samples after austenitising were transferred to a fluidised bed, held for 6 h at 320 °C and then cooled in the air. In the case of the second variant (hereinafter called Br-QT), samples after austenitising were directly quenched in oil, tempered for 1 h at 580 °C and cooled in the air. The batch temperature during the heat treatments was controlled through the control sample with a thermocouple inside. The time of the isothermal segments was counted from the moment the control sample reached a temperature of 5 °C higher (nanobainitising) or lower (tempering) than the nominal temperature. The temperature regime of these segments was ±5 °C. Moreover, the non-borided samples were subjected to the same treatments. The variants of the treatments described in this work are summarised in Table 2. The treatments carried out are schematically shown in Figure 2.

### 2.4. Microstructural Investigation

The metallographic observations were carried out on wear test cylindrical sample (rollers) cross-sections. After mechanical grinding and polishing, the chemical etching with 4% Nital reagent was conducted to reveal the microstructure. The microstructural zones (borides, porous, silicon-rich ferrite) in the borided layer were determined based on an average of 30 measurements.

The TEM observations were performed using the samples cut from rollers cores. They were then cut into thin slices, ground to a thickness of about 100 μm and electrolytically thinned until their perforation. Observations were carried out with the transmission electron microscope TEM JEOL 1200 EX II (Tokyo, Japan). The thicknesses of bainitic ferrite plates and austenite layers were determined based on measurements from 30 randomly selected places. The measured values were divided by π/2, according to the methodology described in the work of Garcia-Mateo et al. [28].

To estimate the silicon and carbon concentration profile across the borided layer, the glow–discharge optical emission spectroscopy (GDOES) technique was used. The examination was performed on a borided disc with the LECO GDS 850a spectrometer (Saint Joseph, MI, USA).

The phase composition of the borided disc layer was determined through X-ray diffraction (XRD). Measurements were carried out on a Rigaku SmartLab 3kW diffractometer (Tokyo, Japan). For the apparatus, the radiation source was a Cu tube with operating parameters of U = 40 kV and I = 30 mA. The Bragg–Brentano θ/2θ measuring geometry was used with a measuring step of 0.02°.

Magnetic tests were carried out to determine the amount of retained austenite in the steel core after heat treatments. This method uses differences in magnetic properties of phases existing in steel (ferromagnetic ferrite and paramagnetic austenite). During the examination, saturation magnetisation for the test material and the standard were compared. Samples in the form of round slices (ca. 1 mm thick, with a diameter of ca. 2.8 mm), which were obtained from rollers cores, were used.

### 2.5. Examination of the Tribological and Mechanical Properties

The Vickers microhardness HV0.05 measurements were performed on mechanically ground and polished borided rollers cross-sections using the Future-Tech FM-810 tester (Kawasaki City, Japan). The indents’ sizes were measured using the Keyence VHX 7000 light microscope (Osaka, Japan). Each value in the hardness distribution graph was the average of the 3 measurements. The hardness of the core was an average of 9 measurements.

**Figure 3:**
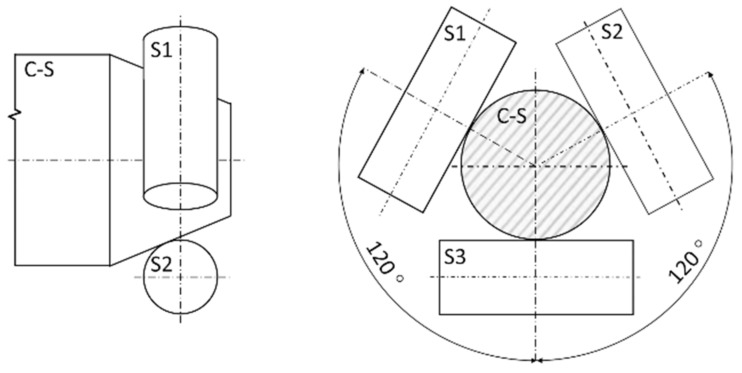
Scheme of “three rollers—taper” method setup: counter-specimen (C-S) and rollers (S1, S2, S3) according to the Polish standard PN-83/H-04302.

**Figure 4:**
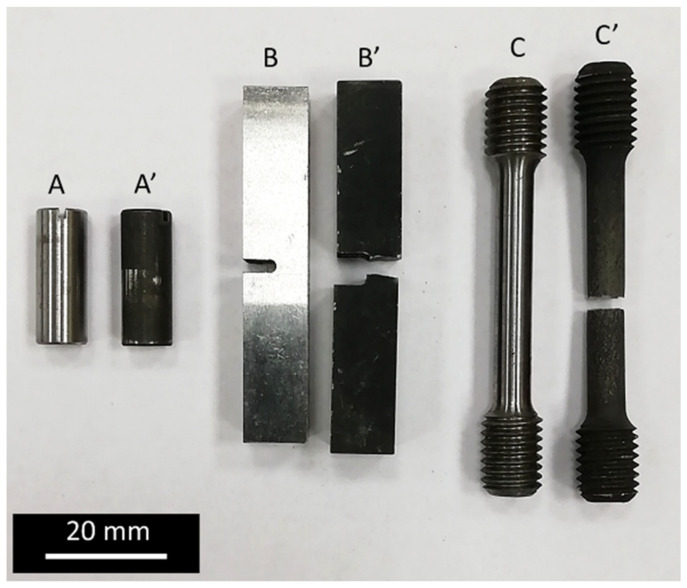
Untreated specimens for examination of tribological (A) and mechanical properties (B, C) and their analogue after experiments (A′, B′, C′).

To examine the frictional wear resistance, the “three rollers—taper” method was applied according to the Polish standard PN-83/H-04302. During tests, rollers (22 mm long, with a diameter of 8 mm) were rubbed against a conical surface of counter-specimen (apex angle of 45°) made of heat-treated EN C45 steel (30HRC) under constant unit loads of 200 and 400 MPa. The oil LUX 10 was used as a lubricant. A measure of linear wear was the wear depth, which was determined by gauging the diameters of the ellipses formed on the surface of each roller and averaging them. Worn surfaces were analysed using the Hitachi S-3500 scanning electron microscope (Tokyo, Japan) in secondary electron mode (SE). The set-up for this method is schematically shown in Figure 3, while the specimen is pictured in Figure 4.

The static tensile test was performed on cylindrical samples (Figure 4) with the MTS810 servo-hydraulic machine. The test was carried out with a constant elongation rate of 0.036 mm/s. An MTS extensometer with a gauge length of 25 mm was used to measure the elongation of the sample. For each treatment variant examination, 3 samples were tested and the results were averaged. The borided samples had the same geometry as the non-borided ones and differed from them through the presence of a boride layer on the entire surface. Fracture surfaces of specimens used in tensile tests were investigated with the Nikon SMZ1270 stereo microscope (China).

Impact toughness tests were performed on the standard 10 × 10 × 55 mm rectangular samples (U-shaped notch, Figure 4) with a Charpy Zwick hammer (initial hammer energy of 300 J). For each treatment variant examination, 3 samples were tested and the results were averaged. The borided samples had the same geometry as the non-borided samples and differed from them through the presence of a boride layer on the entire surface (also on the notch surface).

## 3. Results and Discussion

### 3.1. Microstructure and Microhardness

**Figure 5:**
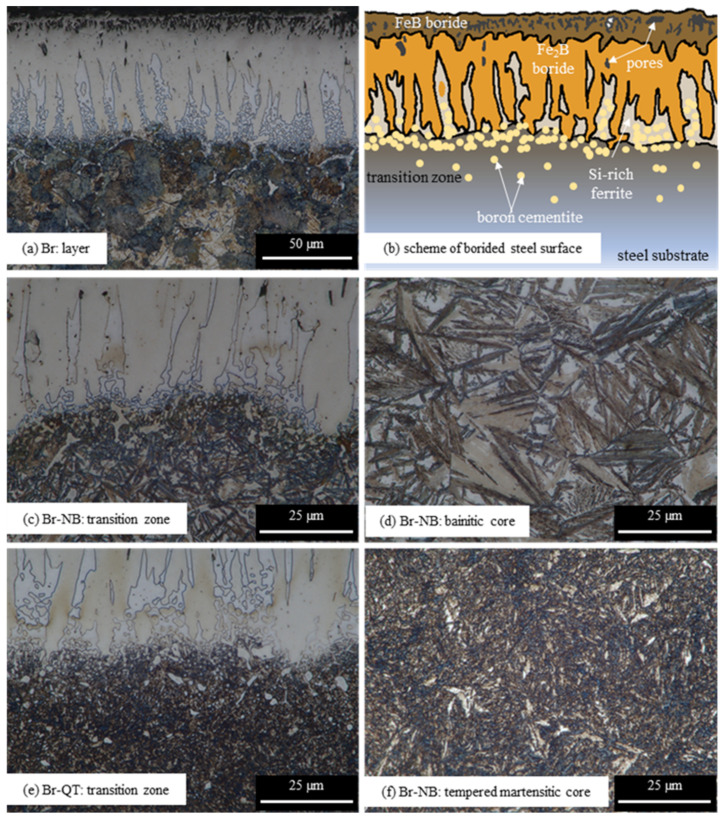
Microstructures of layer, neighbour substrate and core of borided steel: (**a**) initial state Br—without further heat treatment, (**b**) scheme of borided steel surface, (**c**,**d**) after nanobainitising Br-NB and (**e**,**f**) after quenching and tempering Br-QT.

**Figure 6:**
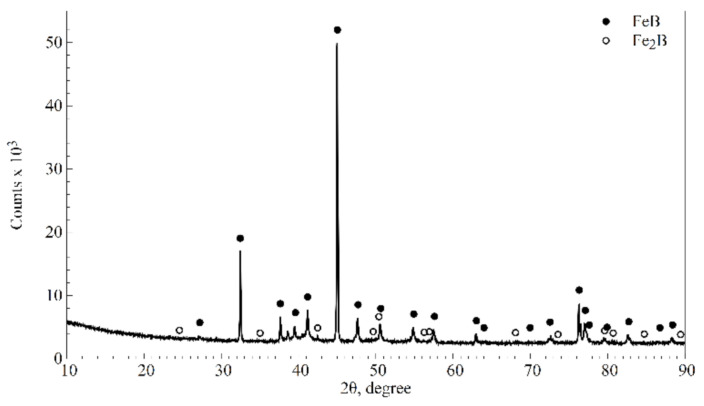
X-ray diffraction patterns of borided steel at 900 °C for 4 h.

The starting material for both examined heat treatment variants (nanobainitising or quenching and tempering) was EN 66SiMnCrMo6-6-4 bearing steel, pack-borided for 4 h at 900 °C. The microstructure of the as-borided steel surface (hereinafter variant Br) without further heat treatment is shown in Figure 5a. It is also schematically presented in Figure 5b. The presence of phase contrast indicates that both FeB (dark pink areas, rich in pores) and Fe_2_B (light pink areas) boride types are present. Joshi and Hosmani [9] and Motallebzadeh et al. [29] reported similar phase contrast between FeB and Fe_2_B borides; however, the FeB zone was broader and easier to detect in their works. XRD analysis confirms the presence of two types of borides on the borided layer surface (Figure 6).

**Figure 7:**
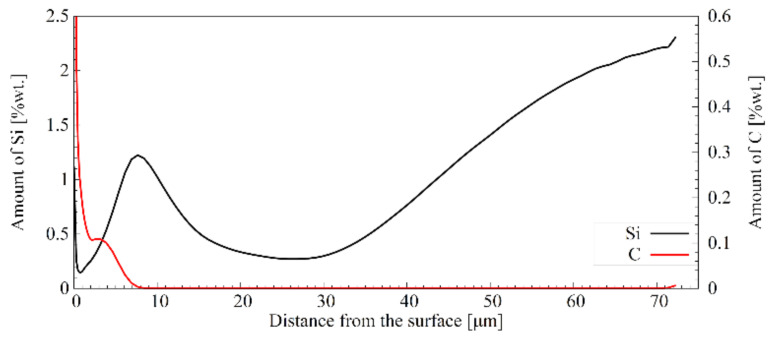
Distribution of silicon and carbon in borided layer.

The white phase separating the borides’ needles (Figure 5a) was recognised as silicon-rich ferrite. Investigation results using the GDOES method indicate the redistribution of silicon atoms in a borided layer (Figure 7). Silicon concentration in the examined steel is 1.63 wt%. As can be seen, the area occupied by borides exhibits reduced silicon content. However, with the appearance of silicon-rich ferrite, the amount of this element increases. The silicon amount is significantly higher in the area dominated by silicon-rich ferrite than in the sample core. The presence of this phase is a typical phenomenon in borided steels. Since silicon atoms hardly dissolve in the crystal lattice of borides, they are pushed by the front of new phase growth [27,30]. Silicon atoms redistribution leads to developing areas with a high silicon concentration that prevents the borides’ growth. Silicon-rich zones are a natural consequence of the chemical composition of the steel intended for nanobainitising (with more than 1 wt% of Si) and can form even if silicon content is relatively small [31,32]. The presence of silicon-rich ferrite is undesirable in the boride layers. However, reducing the silicon-rich ferrite amount beneath iron borides would be difficult because of the significant silicon concentration (1.63 wt%) in 66SiMnCrMo6-6-4 bearing steel. The boron diffusion front usually pushes the silicon in a core direction. Simultaneously, the same effect was observed in the case of carbon, which does not dissolve in borides. Hence, the silicon-rich ferrite will always appear in the transition zone. The longer the boriding time, the greater the amount of this phase that will be observed. This phase can be eliminated if a material with an extremely low silicon content is borided.

Several microstructural zones can be distinguished when analysing the microstructure of the as-borided surface (Figure 5a,b). Just below the surface, there is a porous zone. It is located at a depth of ca. 11.2 ± 2.1 µm. The voids visible in the zone below are probably the effect of crushing out silicon-rich ferrite fragments. The porosity is typical of the boride layers. The Kirkendall effect is often evoked to explain the origin of porosity: differences in the diffusion coefficients of boron, iron and alloying elements promote the formation of voids [33,34]. The porous zone close to the surface was often observed when boriding low-alloy steels with a relatively high carbon content, as well as after hybrid treatment consisting of pre-carburising or pre-carbonitriding and boriding [14]. It could result from the intensive diminishing of the carbon content in the zone close to the surface. Simultaneously, the impact of the boriding atmosphere on the surface during pack boriding is not fully understood. It seems that gas boriding in N_2_-H_2_-BCl_3_ atmosphere could limit the porosity of the zone close to the surface in high-carbon steels [35]. Besides shallow porosity, boride columns grow densely to a depth of ca. 29.7 ± 7.1 µm. Below this area, the areas of silicon-rich ferrite appear and start to separate borides. At depths from 53.3 ± 5.3 µm, globular precipitations believed to be boron cementite can be seen [30]. They appear in the area of silicon-rich ferrite and enter the substrate material. Their presence is associated with pushing carbon atoms towards the substrate by the growing layer of borides (Figure 7). The layer’s interface is distinct and mildly wavy. The formed layers have a thickness of 70.1 ± 4.9 µm. Similar borided layers microstructures were revealed in other works [16,27,31,32]. Below is the substrate of a layer with a martensitic structure resulting from the cooling conditions after boriding. Moreover, a transition zone is visible in the substrate and is clearly pearlitic (Figure 5a). Its occurrence can be explained by the redistribution of carbon atoms pushed away by the growing borides (Figure 7) [30].

**Figure 8:**
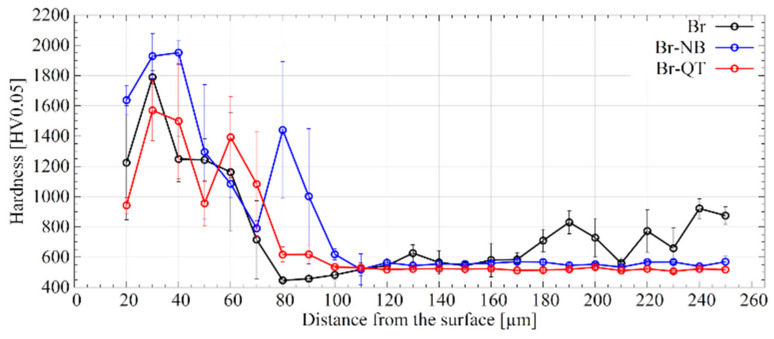
Microhardness distribution in borided steel surface for different post-treatment variants.

According to Krukovitch, the FeB borides exhibit a hardness of 1760–2340 HV and Fe_2_B borides of 1260–1960 HV [4], depending on the steel’s chemical composition. The average microhardness for the Br variant is within this range (Figure 8). However, a significant spread of results can be observed. Undoubtedly, this is due to the porosity of the layer and the presence of silicon-rich ferrite, which do not provide sufficient mechanical support during indentation. The presence of silicon-rich ferrite also explains the high standard deviation values for measurements up to a depth of ca. 90 µm. Beneath 80 µm, i.e., in the transition zone, the microhardness decreases sharply. There is a microhardness pit in the silicon-rich ferrite region [36]. A similar effect was observed in other studies [26,37,38]. In the specimen’s core, the average microhardness value is maintained at ca. 600 HV0.05. Moreover, the microhardness values are greatly dispersed in the core, which results from the martensitic–pearlitic microstructure.

Heat treatment subsequent to boriding modifies the microhardness distribution in the layer and the core. Up to a depth of 70 µm, microhardness can be considered similar for variants Br, Br-NB and Br-QT. The highest measured microhardness value for Br-NB was 2103 HV0.05 at a depth of 30 µm. For depth between 70 and 110 µm, microhardness increases for the variants Br-NB and Br-QT compared to variant Br. Undoubtedly, the strengthening of this area is the effect of post-boriding heat treatment. In the case of the Br-NB variant, microhardness increases at a depth of 80–90 µm, while the standard deviation is also high at that depth. This effect can be explained by slightly deeper penetration of the boride columns into the steel core. In the core area, the microhardness of the variants Br-NB and Br-QT remains at a similar level of ca. 520–560 HV0.05.

**Figure 9:**
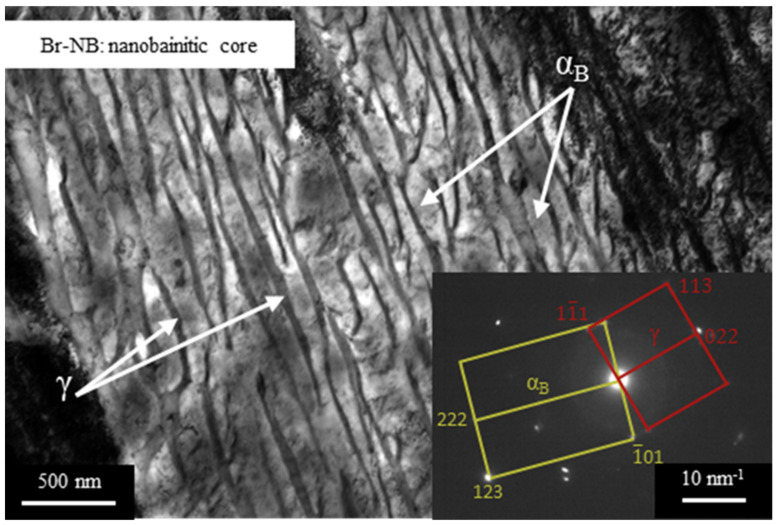
TEM microstructures of steel core after variant Br-NB heat treatment. Nanobainitic areas consisting of bainitic ferrite plates (light, α_B_) and thin layers of residual austenite (dark, γ) can be observed.

Understandably, the microstructures of transition zones and cores differ for these variants. The core’s microstructure for the variant Br-QT consists of tempered martensite, retained austenite and carbides (Figure 5e,f). According to magnetic measurements, the amount of retained austenite in the core is 9.2 ± 2.1 vol%. In contrast, the Br-NB core microstructure consists of bainitic ferrite and retained austenite (Figure 5c,d). Thin and long sheaves of bainitic ferrite (darker components) are visible, which separate relatively small blocks of residual austenite (lighter components). According to magnetic measurements, the amount of retained austenite in the core is 30.5 ± 1.8 vol%. TEM observations revealed that the nanobainitising led to the formation of nanobainitic areas (Figure 9). These areas are composed of bainitic ferrite plates 61 ± 16 nm thick (bright constituents), separated by a residual austenite film 36 ± 17 nm thick (dark components). Previously, the nanobainite in steel with a similar chemical composition was obtained by Dworecka et al. [39].

### 3.2. Wear Resistance

**Figure 10:**
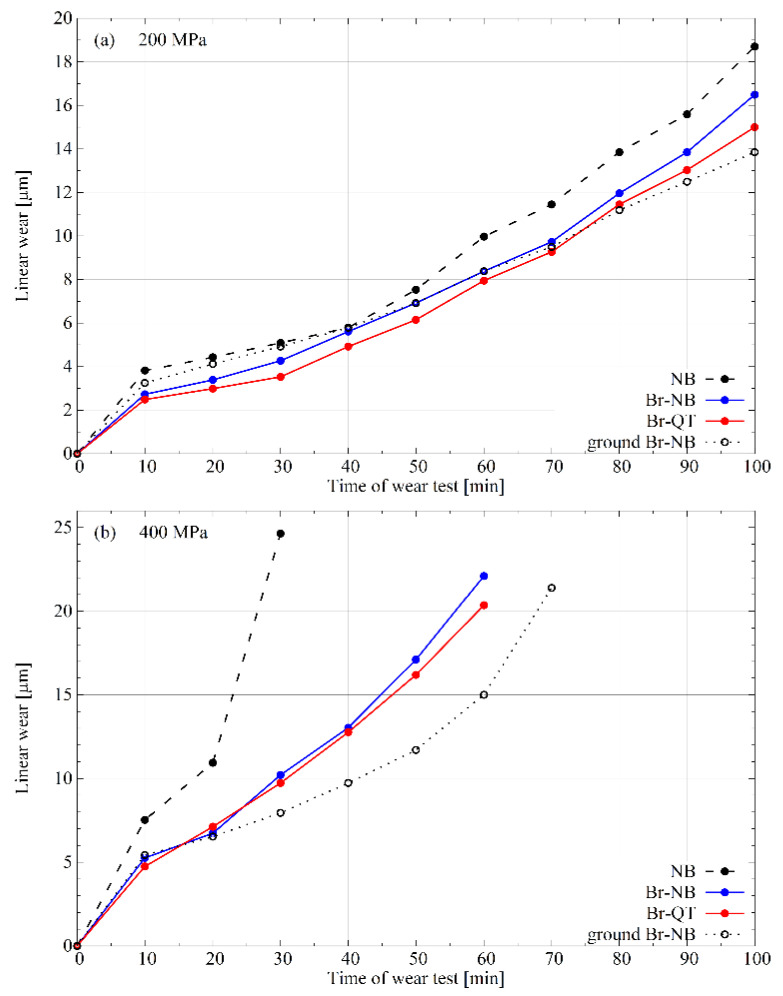
Wear resistance curves for different treatment variants under unit loads of (**a**) 200 and (**b**) 400 MPa.

The frictional wear tests indicated that boriding increased wear resistance compared to the nanobainitised variant without prior boriding NB (Figure 10). A reduction in frictional wear was found for both applied unit loads: 200 and 400 MPa. At both applied unit loads, the Br-NB and Br-QT variants show similar performance. The test results can be analysed concerning the microstructure (Figure 5). It can be noticed that the wear on the borided layers crossed the porous zone. However, the abrasion did not reach the depth where silicon-rich ferrite occurs. Mechanical removal of the porous region for the thickness of ca. 15 µm led to improved wear resistance (ground Br-NB variant). This effect is evident under a unit load of 400 MPa. These results are similar to those reported by Wierzchoń et al. [12].

**Figure 11:**
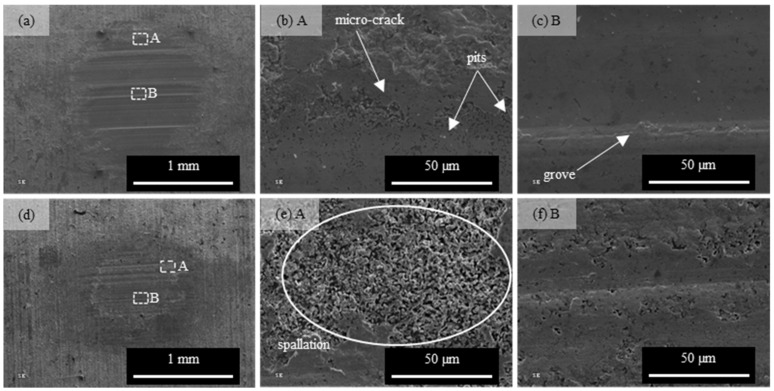
SEM images of wear tracks after “3 rollers—taper” tests under unit load of 400 MPa for heat treatment variants (**a**–**c**) Br-NB and (**d**–**f**) Br-QT.

SEM images of wear tracks are shown in Figure 11. As can be seen, there are identical types of damage for the variants Br-NB and Br-QT. Observations revealed cracks, spallations, pits and groves. The appearance of worn areas indicates a repeated cycle of erosion smoothing, delamination of layer fragments and exposure of new irregular surfaces. A similar character of wear, though resulting from different wear test methods, was also observed by other researchers [11,40,41].

The boride layer thickness, as well as the microstructure of the substrate, significantly influenced the tribological properties of the bearing steel used. Nevertheless, the proposed hybrid treatment is the first attempt to use nanobainitising after boriding to improve the tribological properties of the steel used. The experiment was designed to compare the effect of post-boriding treatment, i.e., conventional hardening and tempering with nanobainitising on the properties of the steel used. The results have shown that nanobainitising could be a beneficial alternative to previously used heat treatments after boriding and that boriding preceding nanobainitising did not prevent the obtaining of nanobainite. Optimising the thickness and microstructure of the boride layer, as well as the transition zone, seemed to be very important for improving the properties of bearing steel. It will be considered as a direction for further research.

### 3.3. Interaction of Layer and Core under Tensile and Impact Loads

**Figure 12:**
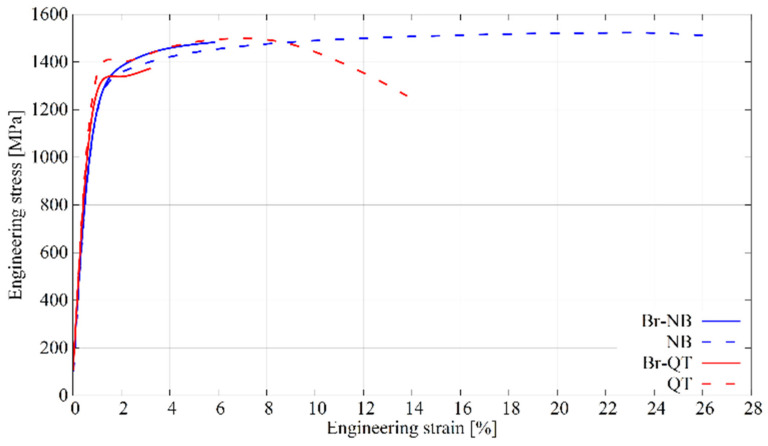
Tensile tests curves obtained for different heat treatment variants.

A static tensile test provides apparent differences between the Br-NB and Br-QT variants. Before considering the differences between hybrid treatments, knowledge of the material’s properties without a layer is crucial. The effect of the heat treatments themselves can be seen by comparing the tensile curves for the variants NB and QT (Figure 12). Nanobainitising allows obtaining of a material with similar strength and much greater plasticity than conventional quenching and tempering treatment. In addition, there is no upper or lower yield point for variant NB as is the case for QT treatment. Steel after NB treatment owes its advantageous properties to its nanocrystalline structure and a high amount of retained austenite. Retained austenite determines the TRIP effect’s occurrence, which ensures high ductility and resistance to cracking in steel. The TRIP effect refers to the transformation of residual austenite into martensite under the influence of stresses or strains. The transformation is possible if the temperature T at which the austenite experiences stimuli meets the following condition: M_s_ < T < M_d_, where M_s_ is the temperature below which the martensitic transformation is thermally induced and M_d_ is the temperature above which the austenite is mechanically stable [42]. At lower temperatures in this range, the generated stresses trigger the martensitic transformation at pre-existing nucleation sites. At higher temperatures, the martensitic transformation is initiated from new nucleation sites produced via slip [42]. The TRIP effect in nanobainitised steels is well-known and has been reported by various researchers [43,44,45,46].

The values determined during the static tensile test are collected in Table 3. As can be seen, the states NB and QT show similar tensile strength but differ in yield strength value. For variant QT, it is about 250 MPa higher than for NB. The lower yield strength results from the higher amount of residual austenite after nanobainitising. On the other hand, the high austenite content leads to an almost four-fold uniform elongation and an almost two-fold total elongation.

**Figure 13:**
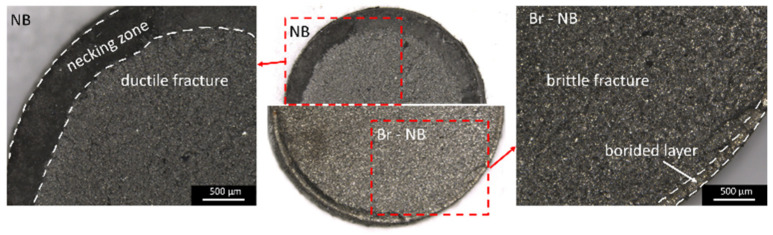
Fracture surfaces after tensile tests of NB and Br-NB variants.

The steel cores formed after the same heat treatments (Br-NB and NB, Br-QT and QT) can be expected to exhibit similar mechanical behaviour. A prior boriding process could then explain possible differences in mechanical properties. Changes in the material caused via pack-boriding include layer formation, redistribution of elements in the transition zone and the effect of the thermal cycle on the microstructure. Of these changes, the formation of the layer is the most radical change brought through the boriding process. Borided layers are remarkably harder than nanobainite but very brittle in comparison. When the material undergoes deformation, the presence of brittle borides promotes crack initiation. Therefore, in the case of Br-NB and Br-QT variants the samples broke up during the static tensile test at lower elongations than for the corresponding non-borided variants. Notably, tensile stresses are generated perpendicularly to the borides columns during the tensile tests. The unfavourable stress system, the presence of porosity and a large amount of silicon-reach ferrite separating the borides columns favour the initiation of the cracks and their propagation into the steel core. Figure 13 shows fracture surfaces after tensile tests of NB and Br-NB variants. The specimen of the NB variant experienced relatively high elongation, causing a reduction in the cross-section. It deformed during the tensile test with neck formation and final fracture in ductile mode. The presence of a boride layer changed the fracture character during the tensile test for the Br-NB variant’s specimen. The Br-NB specimen broke in a brittle manner, firstly in the boride layer and then in the nanobainitic core, which should remain ductile. The initial cracks in the boride layer acted as structural notches, making cracks easy to propagate in the core.

Comparing the Br-NB and Br-QT variants, it can be seen that borided steel shows higher resistance to cracking after nanobainitising. This difference can be explained by the effect of smaller thermal stresses generated during bainitic isothermal quenching in the Br-NB variant than those generated during direct martensitic quenching in the Br-QT variant. On the other hand, the differences in the microstructure and the austenite content between states should be considered. In the case of the Br-QT variant, a zone with a microstructure composed of tempered martensite, retained austenite and carbides extends below the layer (Figure 5e,f). The martensitic needles formed during the quenching cross the parent austenite grain. Their length is limited by the boundaries of the parent phase grain and the previously developed needles. Due to the nature of the bainitic transformation, the microstructure formed during nanobainitising is highly refined. As in the case of martensitic transformation, the bainitic ferrite plates nucleate at the boundaries of the parent austenite grains and grow inside them. A significant difference is that bainitic ferrite has the form of small plates rather than long needles [20]. The bainitic ferrite sheave is not formed by the rapid shear of the austenite crystal lattice. Instead, it develops through the successive autocatalytic growth of subsequent bainitic ferrite plates. The relatively slow kinetics of the transformation allows the development of bainitic ferrite sheaves from different directions. The formed sheaves can provide new heterogeneous nucleation sites for bainitic ferrite, introducing new growth directions. Interlocking bainitic sheaves often strongly fragment the parent austenite grains (Figure 5d). Fragmentation also occurs at a deeper level, where bainitic ferrite plates are surrounded by thin austenite layers (Figure 9). Such a structure effectively makes the crack propagation path longer. In addition, a percolation of austenite impedes cracks propagation [47]. As Kumar and Singh show, the increased fraction of retained austenite in the microstructure increases the toughness (impact and fracture) and ductility of nanobainitic steel [48]. Under stress or strain conditions, retained austenite can transform into martensite. These effects should be considered when looking for the causes of higher cracking resistance in the Br-NB variant than Br-QT.

The impact toughness tests (Table 3) indicate a slight impact toughness difference between the Br-NB and Br-QT variants. However, it should be noted that under impact conditions, the presence of a borided layer regulates the resistance to cracking. Comparing the pre-borided variants (Br-NB, Br-QT) with the corresponding non-borided treatments (NB, QT) reveals how a boride layer strongly weakens the material’s impact toughness. In the case of a developed fracture, the core material can only slightly counteract its further propagation. However, in the case of the Br-NB variant the substrate exhibits higher resistance to impact cracking.

### 3.4. Directions for Hybrid Treatments Optimisation

The research conducted shows a new quality that can be obtained thanks to the combination of surface boriding and nanobainitising of the steel core. The developed hybrid treatment combines the advantages of borided layers and nanobainite. Although the obtained properties can be considered advantageous, it is advisable to optimise the treatment. Two potential areas of development can be identified. The first is to optimise the layer’s properties by modifying its structure. The optimisation should concern reducing layer porosity, tailoring the appropriate thickness, eliminating FeB phase and limiting the silicon-rich ferrite amount. This can be achieved by modifying the boriding conditions and using other methods besides pack boriding. The second direction relates to the optimisation of post-boriding treatments. Since prior boriding leads to the redistribution of atoms in the transition zone, the kinetics of phase transitions in this region also changes. The nanobainitising presented in this paper was designed for the core. It can be expected that optimisation of the treatment, considering the transition zone changes, could improve the interaction between the layer and the substrate.

## 4. Conclusions

1. The developed hybrid treatment, which combines pack-boriding with nanobainitising through the isothermal quenching process, investigated on the EN 66SiMnCrMo6-6-4 bearing steel produces new material with hard and wear-resistant iron boride surface layers and highly strengthened and a relatively plastic nanobainitic core. The needle structure layer produced at 900 °C by 4 h, which is about 70 µm thick, is composed of both FeB in the surface vicinity and Fe_2_B type borides in the layer’s deeper areas. Between the Fe_2_B needles, the areas of silicon-rich ferrite form. The boride layer growth results in the formation of silicon- and carbon-enriched transition zone in the steel substrate. Properly selecting bainitising parameters for the previously borided steel leads to the carbide-free nanobainite in the steel core. However, the modified chemical composition in the transition zone makes the substrate’s microstructure in the layer’s neighbourhood highly refined compared to the core.
2. The formation of the boride layer on the EN 66SiMnCrMo6-6-4 bearing steel with nanobainitic core results in a significant surface hardness increase from ca. 600 up to ca. 2000 HV0.05. In the as-borided steel in the substrate region situated in the vicinity of the layer, hardness is visibly lower than in the core due to the chemical composition modification induced through the growing layer. Due to post-boriding bainitising treatment, the similar hardness level in the substrate region neighbouring with the boride zone is restored and the hardness is stabilised in the transition zone compared to the as-borided state.
3. The hard boride layer produced via the hybrid process on the EN 66SiMnCrMo6-6-4 bearing steel with nanobainitic core results in a significant increase in wear resistance, especially under higher unit loads examined via the “three rollers—taper” method. The wear resistance of the borided steel subjected to post-boriding nanobainitising treatment is similar to steel properties after post-boriding conventional quenching and tempering treatment with slightly better performance for the classic variant. As demonstrated, further wear resistance improvement can be achieved via the mechanical removal of the porous boride layer zone.
4. The mechanical tests revealed that the borided layer, which is hard but susceptible to cracking, reduces the strength, ductility and impact toughness under tensile and impact load conditions. However, it was found that due to its unique nanobainitic core microstructure, borided and nanobainitised steel exhibits a crucial advantage of much higher rupture strength and ductility (1481 MPa and 5.38% versus 1376 MPa and 2.59%) and slightly better impact toughness (7.5 J/cm^2^ versus 6.5 J/cm^2^) than steel after post-boriding quenching and tempering treatment. This effect is supposed to be related to more difficult crack spreading from the boride layer into the nanobainitic core, which is composed of strengthened carbide-free bainitic plates and ductile retained austenite layers.

## Figures and Tables

**Table 1:** Chemical compositions of EN 66SiMnCrMo6-6-4 steel (in wt%).

C	Si	Mn	Cr	Mo	Ni	Fe
0.68	1.63	1.16	1.03	0.22	0.15	balance

**Table 2:** Parameters and designations of performed heat treatments.

Designation	Boriding	Austenitising	Quenching	Tempering
Br	900 °C 4 h	-	-	-
Br-NB		925–935 °C 15 min	isothermal 320 °C 6 h	-
Br-QT			direct to RT	580 °C 1 h
NB	-		isothermal 320 °C 6 h	-
QT	-		direct to RT	580 °C 1 h

**Table 3:** Mechanical properties obtained for different treatment variants. Ultimate tensile strength (UTS) is given for non-borided variants and rupture strength (RS) is given for borided variants.

Variant	UTS or RS [MPa]	Yield Strength R_p0.2_ [MPa]	Uniform Elongation [%]	Total Elongation [%]	Impact Toughness KU [J/cm^2^]
NB	1530 ± 3	1038 ± 26	21.82 ± 0.52	25.96 ± 0.40	48.3 ± 2.1
QT	1490 ± 8	1288 ± 7	6.5 ± 0.24	12.92 ± 0.64	20.0 ± 2.2
Br-NB	1481 ± 1	1003 ± 25	-	5.38 ± 0.41	7.5 ± 0.7
Br-QT	1376 ± 3	1222 ± 4	-	2.59 ± 0.22	6.5 ± 0.3

## Data Availability

All data included in this study are available upon request by contacting the corresponding author.
